# Outcome evaluation of the zero-profile device comprising two integrated variable angle screws used for single-level cervical degenerative disc disease: comparison with the plate-cage construct

**DOI:** 10.3389/fsurg.2026.1800991

**Published:** 2026-04-15

**Authors:** Can Cao, Yun-Sheng Wang, You-bin Yang, Xian-Da Gao, Xing-Zhu Xu, Qing-Tao Liu, Lin-Feng Wang

**Affiliations:** 1 Department of Spinal Surgery, The Third Hospital of Hebei Medical University, Shijiazhuang, Hebei, China; 2 Department of Orthopedics, Hebei General Hospital, Shijiazhuang, Hebei, China; 3 Department of Orthopedic Surgery, The First Hospital of Hebei Medical University, Shijiazhuang, Hebei, China

**Keywords:** anterior cervical discectomy and fusion, segmental kyphosis, segmentalangle, variable angle screw, zero-P VA

## Abstract

**Background:**

Zero-P VA device is a unique zero-profile device comprising only two integrated variable-angle screws which may provide inferior mechanical stability compared with other types of devices. There is a lack of comprehensive clinical and radiological evidence comparing the plate-cage construct (PCC) and the Zero-P VA device in single-level anterior cervical discectomy and fusion (ACDF).

**Methods:**

We retrospectively reviewed consecutive patients who underwent single-level ACDF using either the Zero-P VA device (50 cases) or the PCC (51 cases). Clinical outcomes were evaluated using the Visual Analogue Scale (VAS) and Japanese Orthopaedic Association (JOA) scores. Radiological outcomes were assessed using standard lateral cervical x-ray films. Data were recorded preoperatively, immediately postoperatively, and at 3- and 12-month follow-up visits.

**Results:**

VAS and JOA scores, cervical alignment, segmental angle, and surgical segment height were all significantly improved postoperatively in both groups. However, in the Zero-P VA group, the segmental angle and anterior height of the surgical segment at 3 and 12 months decreased significantly compared with immediate postoperative values and were significantly lower than those observed in the PCC group. The rate of segmental kyphosis was significantly higher in the Zero-P VA group at the 12-month follow-up (12% vs. 0%, *p* < 0.05), while fusion rates were comparable.

**Conclusion:**

The Zero-P VA device provides short-term clinical outcomes comparable to those of the PCC for single-level ACDF. However, it is associated with inferior radiological outcomes, specifically greater loss of segmental lordosis and anterior surgical segment height. The surgeon's choice of implant therefore involves a clinical trade-off between the established surgical advantages of a zero-profile system and the superior radiological stability offered by the PCC.

## 1 Introduction

Cervical degenerative disc disease is a common condition in adults, often manifested as neck pain, radiculopathy, and myelopathy, which seriously affects patients' health and quality of life (1). Surgical treatment should be considered for patients who are refractory to standardized conservative management (1, 2). Anterior cervical discectomy and fusion (ACDF) has evolved over more than 60 years and has undergone continuous improvement (2, 3). From bone grafting alone to the addition of plate-screw fixation, ACDF has become the most commonly used surgical method for treating cervical spondylotic myelopathy and radiculopathy due to its direct anterior decompression, interbody fusion, and lower complication rates compared with laminectomy (2). With its successful application in cervical trauma in the early 1980s, the anterior cervical plate was widely adopted to reduce the subsidence rate to treat cervical degenerative disc disease (2). The application of a plate-screw construct with an interbody cage or autograft is now far more common than autograft alone because it can achieve immediate stabilization, better restore lordosis and disc height, and reduce the risk of subsidence (4). Its outcome is quite satisfactory in most cases, but the associated complications can still be significant and occasionally severe, such as postoperative dysphagia, adjacent segment degeneration, dysphonia, esophageal injury, and hematoma (5, 6). There is evidence that these complications are associated with the prolongation of operation time, the expansion of exposure, and the application of the anterior cervical plate (6, 7).

**Figure 1:**
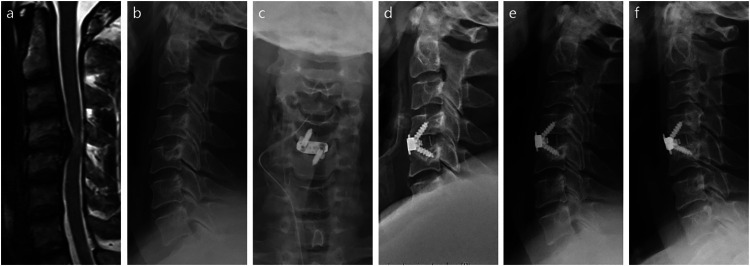
Illustration of the zero-P VA device using images obtained from a 46-year-old male with cervical spondylotic myelopathy who underwent ACDF at C4−5 segment. **(a)** Preoperative sagittal T2-weighted MR image. **(b)** Preoperative lateral x-ray film. **(c)** Immediate postoperative anteroposterior x-ray film. **(d)** Immediate postoperative lateral x-ray film. **(e)** Lateral x-ray film at 3 months post-surgery. **(f)** Lateral x-ray film at 12 months post-surgery.

To mitigate these complications, “zero-profile”, “no-profile”, and “anchored spacer” devices—which combine a polyetheretherketone (PEEK) cage with integrated clips or screws for fixation through the endplates—were introduced and developed (8, 9). These devices can be implanted entirely within the intervertebral space or have a low profile anterior to the vertebral body to reduce surgical exposure and irritation to the esophagus, and have been shown to reduce complications, especially postoperative dysphagia and adjacent segment degeneration (8, 10, 11). While most studies have reported comparable short-term and medium-term clinical outcomes compared with traditional ACDF with plate-screw fixation, their radiological outcomes remain a subject of controversy. The differences in reported radiological outcomes mainly concern cage subsidence and the loss of lordosis, which may be related to long-term clinical outcomes (4, 9, 12–15). The stability of the zero-profile device is still a matter of concern (9, 16). Differences in the number of screws, surgical level, number of surgical levels, implant placement position, implant design, and follow-up period may all contribute to the controversy (9, 12–19). It is speculated that the stability of a zero-profile device with two integrated screws may be inferior to that of devices with four integrated screws (9, 17). In addition, biomechanical research has shown that the variable-angle screw spacer appears to be less stable than the locked-screw spacer (19). Therefore, we sought to explore the clinical and radiological outcomes of ACDF with the Zero-P VA (a midline zero-profile anchored spacer with two integrated variable-angle screws) (Figure 1) in single-level cervical degenerative disc diseases, with particular emphasis on the radiological outcomes, in comparison with the plate-cage construct (PCC). We hypothesized that the stability of the Zero-P VA device may be inferior to that of the PCC, which could affect the postoperative outcome, at least the radiological outcome. To our knowledge, there is limited comprehensive clinical and radiological evidence comparing the PCC and the Zero-P VA device, or any similar device with two integrated variable-angle screws, in single-level ACDF.

## 2 Material and methods

### 2.1 Patient population

This study was approved by the institutional review board of our hospital, and data were retrospectively collected from patients with single-level cervical degenerative disc disease involving C3 and C7 with radiculopathy and/or myelopathy treated with either the Zero-P VA device (DePuy Synthes, Switzerland) or the traditional plate-cage construct (PCC) (WEGO, China) from December 2015 to December 2020. The indications for ACDF included failure of conservative treatment or manifestation of progressive neurological deficits (1). A total of 312 patients underwent single-level ACDF during this period. Based on our inclusion and exclusion criteria, 101 patients were enrolled consecutively in this study. The exclusion criteria were as follows: a history of cervical spine surgery; additional posterior instrumentation; fracture, tumor, or infection; ossification of the posterior longitudinal ligament in multiple segments; diffuse idiopathic skeletal hyperostosis; cervical congenital fusion deformity; neurological diseases, such as amyotrophic lateral sclerosis; incomplete follow-up data; and unmeasurable radiological data. The complete follow-up data were required to include the clinical and radiological outcomes at both 3 and 12 months after surgery. According to our criteria, patients enrolled in this study were divided into two groups: the Zero-P VA group (50 cases) and the PCC group (51 cases). Informed consent was obtained from all patients. Before a single-level ACDF surgery, we will explain the advantages and disadvantages of Zero-P VA and PCC to the patient and their family. For example, PCC has been used for a long time with proven efficacy, but compared to Zero-P VA, it may have disadvantages such as longer surgical time, greater trauma, and a higher incidence of dysphagia and adjacent segment disease (2, 8). However, Zero-P VA is a newly applied device, with the disadvantage that its stability and long-term efficacy are not yet fully established (19). The final choice of internal fixation device will be made by the patient and their family. Preoperative demographic data and clinical patterns of these patients are summarized in Table 1.

**Table 1:** Preoperative demographic data and clinical pattern of the patients.

Terms	Zero-P VA group	PCC group	*p*-value
Patient numbers	50	51	
Mean age (years)	48.82 ± 10.36	49.94 ± 10.31	0.587
Gender (male/female)	29/21	29/22	0.908
Diagnostic classification			
Cervical spondylotic myelopathy	28	33	0.665
Cervical spondylotic radiculopathy	13	11	
Cervical spondylotic myeloradiculopathy	9	7	
Level of surgical segment			
C3–4	3	9	0.225
C4–5	15	14	
C5–6	30	24	
C6–7	1	4	

### 2.2 Surgical technique

All procedures were performed by a team led by a single senior, experienced spine surgeon from our institution. After the induction of general anesthesia, a standard right-sided Smith-Robinson approach was used for exposure. Discectomy, removal of nucleus pulposus fragments and osteophytes, and dissection of the posterior longitudinal ligament were performed at the targeted level, as confirmed by C-arm fluoroscopy, with the aid of a Caspar retractor to achieve thorough decompression of the dura and nerve roots. The anterior osteophytes were removed if present, and the anteroinferior margin of the cranial vertebral body was removed using a rongeur when the surgical field of view was obstructed. The bone obtained during the surgery was reserved for bone grafting. Following endplate preparation using a curette and high-speed burr for bone grafting, the appropriate size of the cage was chosen based on the trial implants and intraoperative fluoroscopy. The cages were filled with autogenous bone or a mixture of autogenous bone and allogeneic bone (when autogenous bone was insufficient), then the Zero-P VA device or the cage of the PCC was inserted. After removal of the Caspar retractor, two screws were used for fixation in the Zero-P VA group, and four screws were used to fix the pre-bent locking plate in the PCC group. Finally, the incision was closed after the placement of a drainage tube. Subsequently, C-arm fluoroscopy was used to confirm the position of the implant, and a negative pressure drain was retained routinely. On the first day after surgery, ambulation was allowed, and a cervical collar was worn for 4 weeks.

### 2.3 Clinical evaluation

Data on age, sex, surgical segment, and diagnosis were collected. The Visual Analogue Scale (VAS) score and the Japanese Orthopaedic Association (JOA) score were used to evaluate clinical outcomes preoperatively and postoperatively at 3 and 12 months. The VAS scores were used to evaluate neck and arm pain caused by cervical spondylotic radiculopathy and cervical spondylotic myeloradiculopathy, while the JOA scores were used to evaluate spinal cord dysfunction caused by cervical spondylotic myelopathy and cervical spondylotic myeloradiculopathy.

### 2.4 Radiological evaluations

**Figure 2:**
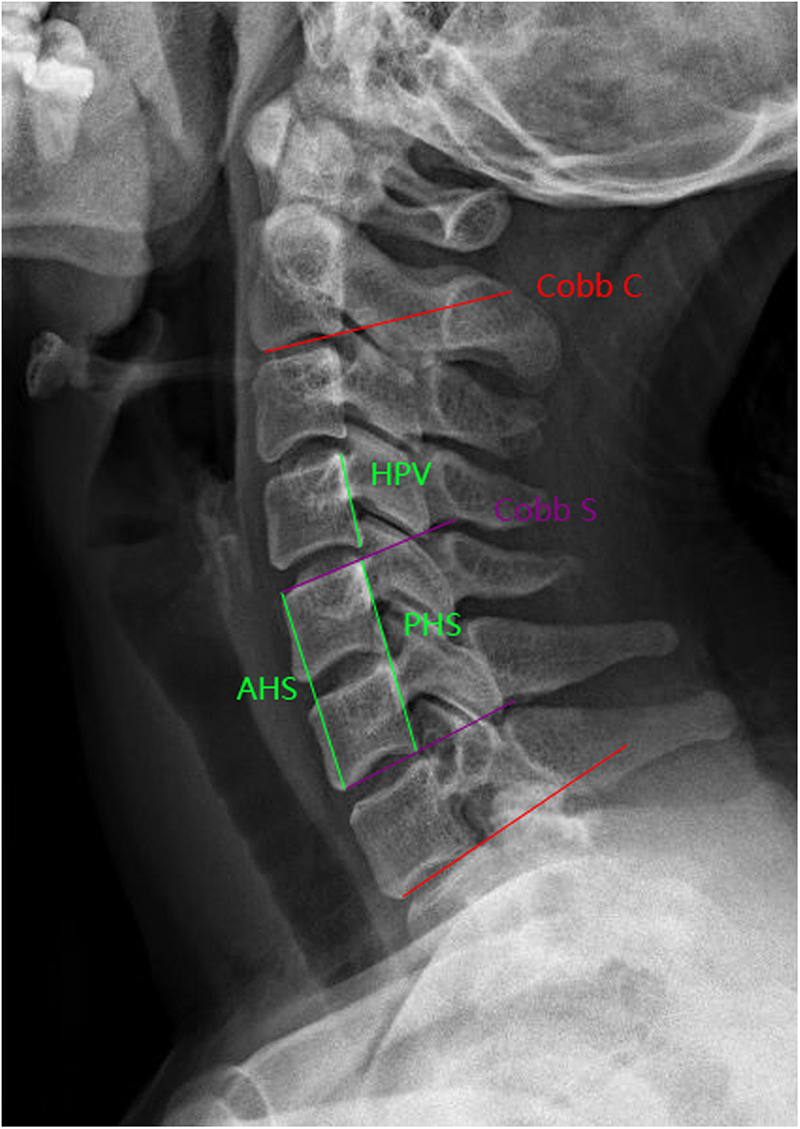
Measurement of Cobb C, Cobb S, AHS, PHS, and HPV via the lateral x-ray film.

Standard cervical lateral x-ray films were obtained preoperatively, and postoperatively at 3 and 12 months in the standing position, while the immediate postoperative cervical lateral x-ray film was obtained in the supine position before transfer to the post-anesthesia care unit. Cervical alignment (Cobb C), segmental angle (Cobb S), and anterior and posterior height of the surgical segment (AHS, PHS) were measured via the lateral x-ray film (Figure 2). Cervical alignment (Cobb C) was assessed using the Cobb angle formed by the lower endplate of C2 and the lower endplate of C7. Since the immediate postoperative x-ray film was obtained in the supine position, Cobb C was not measured at that time point. The segmental angle (Cobb S) was defined as the Cobb angle formed by the upper endplate of the cranial vertebral body and the lower endplate of the caudal vertebral body in the surgical segment. Given the observed differences in Cobb S at 3 and 12 months postoperatively between the two groups, we further evaluated the rate of segmental kyphosis. Consistent with the literature, segmental kyphosis was defined as Cobb S ≤−5 and segmental lordosis as Cobb S >−5 (20, 21). The anterior height of the surgical segment (AHS) was defined as the distance between the anterior edges of the upper endplate of the cranial vertebral body and the lower endplate of the caudal vertebral body within the surgical segment, while the posterior height of the surgical segment (PHS) was defined as the distance between the posterior edges of the upper endplate of the cranial vertebral body and the lower endplate of the caudal vertebral body. To eliminate errors caused by image magnification or reduction, the height of the posterior margin of the vertebral body (HPV) above the surgical segment (or below the surgical segment for C3–4 levels) was also measured (Figure 2). AHS and PHS measured immediately after surgery and at 3 and 12 months postoperatively were corrected based on the preoperative measurements. The calculation method is as follows: corrected postoperative AHS or PHS = postoperative measurement × (preoperative HPV/postoperative HPV). Bone fusion was also evaluated via the lateral x-ray film. Bone fusion was defined as the presence of contiguous bone bridges and the absence of radiolucent gaps between the graft and vertebral endplates in the surgical intervertebral space at 12 months postoperatively (13). Imaging measurements were performed by a spine surgeon who was not involved in the surgery, and the results were reviewed and confirmed by two additional spine surgeons.

### 2.5 Statistical methods

SPSS 23.0 statistical software was used for analysis. Qualitative data were tabulated with frequencies and percentages, and quantitative data were expressed as the mean ± standard deviation (SD). The Chi-square test was used to compare qualitative data between the two groups. The Student's *t*-test or Mann–Whitney *U* test was used to compare quantitative data. Analysis of variance (ANOVA) was used for comparing multiple measurements within a group, followed by Bonferroni's multiple comparison test for pairwise comparisons. A *p*-value < 0.05 was considered statistically significant.

## 3 Results

There were no significant differences in the preoperative demographic data and clinical patterns between the two groups (*p* > 0.05, respectively) (Table 1). The same was true for preoperative VAS score, JOA score, Cobb C, Cobb S, rate of segmental kyphosis, AHS, and PHS (Tables 2–8). All 101 patients completed the operation successfully, and no adverse events related to the surgical treatment were recorded. Within the 12-month follow-up period, none of the 101 patients required revision surgery. Regarding clinical efficacy, both the VAS scores and JOA scores at every follow-up after surgery in the two groups improved significantly compared with preoperative values (*p* < 0.05, respectively) (Tables 2, 3). Additionally, there were no significant differences in JOA scores and VAS scores at each follow-up time point between the two groups (*p* > 0.05, respectively) (Tables 2, 3).

**Table 2:** Changes of preoperative and postoperative VAS scores.

Terms	Preoperatively	3 months postoperatively	12 months postoperatively
Zero-P VA group	4.64 ± 1.29	1.36 ± 0.49*	1.14 ± 0.47*
PCC group	4.94 ± 1.11	1.22 ± 0.43*	1.06 ± 0.42*
*p*-value	0.430	0.344	0.571

* Compared with preoperative value, *p* < 0.05.

**Table 3:** Changes of preoperative and postoperative JOA scores.

Terms	preoperatively	3 months postoperatively	12 months postoperatively
Zero-P VA group	12.97 ± 1.28	14.57 ± 1.04*	16.03 ± 0.76*
PCC group	12.83 ± 1.57	14.53 ± 1.13*	16.08 ± 0.89*
*p*-value	0.440	0.864	0.800

* Compared with preoperative value, *p* < 0.05.

**Table 4:** Changes of preoperative and postoperative Cobb C.

Terms	Preoperatively	3 months postoperatively	12 months postoperatively	*p*-value
Zero-P VA group	12.10 ± 13.20	15.12 ± 10.42*	14.74 ± 9.48*	0.011
PCC group	12.23 ± 13.64	15.96 ± 12.16*	15.61 ± 11.23*	0.007
*p*-value	0.960	0.710	0.676	

* Compared with preoperative value, *p* < 0.05.

**Table 5:** Changes of preoperative and postoperative Cobb S.

Terms	Preoperatively	Immediate postoperatively	3 months postoperatively	12 months postoperatively	*p*-value
Zero-P VA group	0.20 ± 4.79**	6.42 ± 4.49*	2.50 ± 5.21*, **	2.10 ± 5.57*, **	<0.001
PCC group	0.51 ± 5.70**	5.10 ± 4.30*	5.65 ± 4.65*	5.80 ± 4.86*	<0.001
*p*-value	0.768	0.138	0.02	0.001	

* Compared with preoperative value, *p* < 0.05.

** Compared with the immediate postoperative value, *p* < 0.05.

**Table 6:** Changes of preoperative and postoperative AHS (mm).

Terms	Preoperatively	Immediate postoperatively	3 months postoperatively	12 months postoperatively	*p*-value
Zero-P VA group	35.22 ± 2.99**	38.47 ± 3.12*	36.98 ± 3.15*, **	36.63 ± 3.27*, **	<0.001
PCC group	35.40 ± 2.77**	38.66 ± 2.64*	38.35 ± 2.73*	38.22 ± 2.71*	<0.001
*p*-value	0.759	0.743	0.021	0.009	

* Compared with preoperative value. *p* < 0.05.

** Compared with the immediate postoperative value, *p* < 0.05.

**Table 7:** Changes of preoperative and postoperative PHS (mm).

Terms	Preoperatively	Immediate postoperatively	3 months postoperatively	12 months postoperatively	*p*-value
Zero-P VA group	35.13 ± 2.96**	36.52 ± 2.79*	36.25 ± 2.99*	36.06 ± 3.04*	<0.001
PCC group	35.23 ± 2.48**	37.01 ± 2.64**	36.43 ± 2.52*, **	36.23 ± 2.41*, **	<0.001
*p*-value	0.857	0.363	0.746	0.762	

* Compared with preoperative value, *p* < 0.05.

** Compared with the immediate postoperative value, *p* < 0.05.

**Table 8:** Changes of the preoperative and postoperative cases with segmental kyphosis.

Terms	preoperatively		Immediate postoperatively		3 months postoperatively		12 months postoperatively	
	kyphosis	lordosis	kyphosis	lordosis	kyphosis	lordosis	kyphosis	Lordosis
Zero-P VA group (cases)	7	43	0	50	4	46	6	44
PCC group (cases)	10	41	0	51	0	51	0	51
*p*-value	0.451				0.121		0.033	

**Figure 3:**
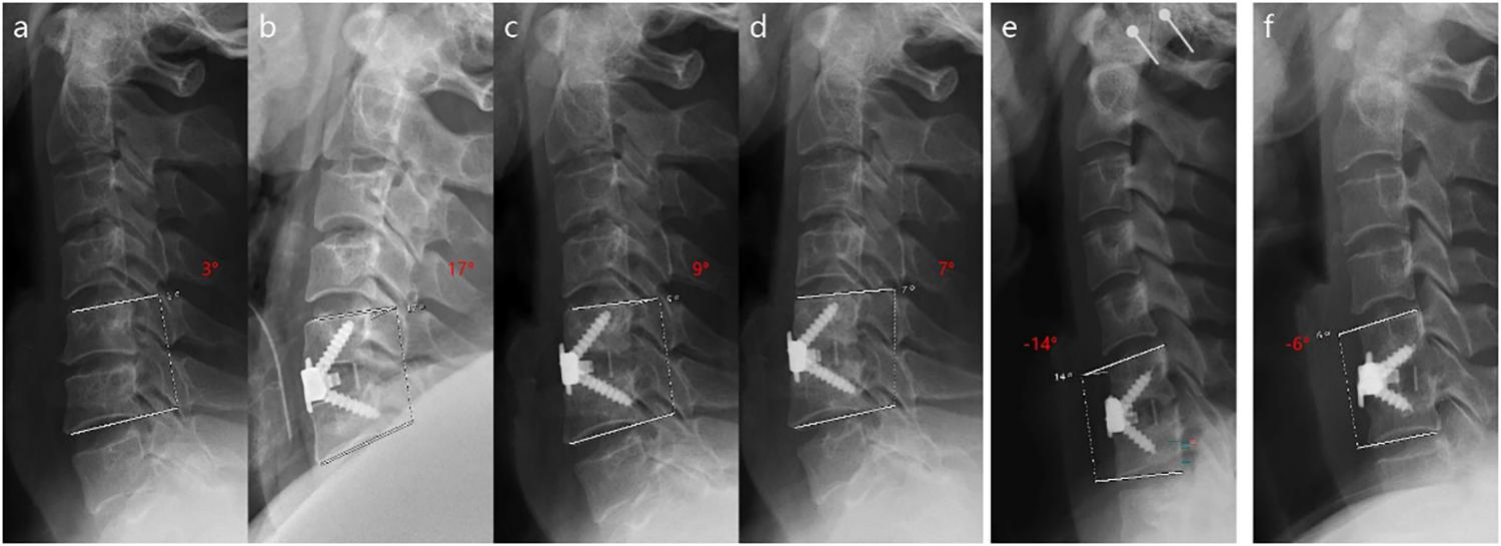
A case showing the loss of the surgically corrected Cobb S. **(a)** Preoperative Cobb S angle was 3°. **(b)** Immediate postoperative Cobb S was 17°. **(c)** The surgically corrected Cobb S was lost to 9° at 3 months post-surgery. **(d)** The surgically corrected Cobb S was further lost to 7° at 12 months post-surgery. **(e,f)** Two cases with different degrees of segmental kyphosis at 12 months post-surgery; the Cobb S values were −14° and −6°, respectively.

Regarding the radiological outcome, all Cobb C, Cobb S, AHS, and PHS measurements at every follow-up after surgery in the two groups increased significantly compared with preoperative values (*p* < 0.05, respectively) (Tables 4–7). In the Zero-P VA group, Cobb S at 3 and 12 months after surgery decreased significantly compared with immediate postoperative values (*p* < 0.05, respectively), whereas in the PCC group, Cobb S at 3 and 12 months increased slightly compared with immediate postoperative values, although the differences were not statistically significant (*p* = 0.108 and *p* = 0.091, respectively) (Table 5). The AHS in the Zero-P VA group and PHS in the PCC group at 3 and 12 months after surgery decreased significantly compared to those immediately after surgery (*p* < 0.05, respectively). In contrast, the AHS in the PCC group and PHS in the Zero-P VA group at 3 and 12 months after surgery showed no significant differences from those immediately after surgery (*p* > 0.05, respectively) (Tables 6, 7). As for the comparison between groups, there were no significant differences in Cobb C and PHS at each follow-up time point or in Cobb S and AHS immediately after surgery between the two groups (*p* > 0.05, respectively) (Tables 4–7). However, the Cobb S at 3 and 12 months after surgery in the Zero-P VA group was significantly smaller than in the PCC group (*p* < 0.05, respectively) (Table 5). Similarly, the AHS at 3 and 12 months after surgery in the Zero-P VA group was significantly lower than in the PCC group (*p* < 0.05, respectively) (Table 6). The rate of segmental kyphosis at 12 months after surgery in the Zero-P VA group was significantly greater than that in the PCC group (12% vs. 0%, *p* < 0.05), while the rates were comparable before surgery, immediately after surgery and at 3 months after surgery (*p* > 0.05, respectively) (Table 8). In the Zero-P VA group, there were no statistically significant differences in VAS scores (1.00 ± 0.00 vs. 1.16 ± 1.58, *p* = 0.718) or JOA scores (15.80 ± 0.84 vs. 16.03 ± 0.78, *p* = 0.531) between the patients with kyphosis and those with lordosis at 12 months after surgery. Additionally, none of the 6 patients with kyphosis reported worsening pain or deterioration of spinal cord function during the follow-up period. A typical case illustrating the loss of the surgically corrected Cobb S, as well as representative cases of postoperative segmental kyphosis are illustrated in Figure 3. The fusion rate of the Zero-P VA group was 98% (49/50), while that of the PCC group was 96.08% (49/51); the difference between the two groups was not statistically significant (*p* = 1.00).

## 4 Discussion

Since ACDF was first introduced in the 1950s for cervical degenerative disc disease by Smith and Robinson, 3 gradual improvements have been made to achieve more satisfactory outcomes (2, 22). An additional anterior cervical plate, developed in the 1980s, was shown to result in a more lordotic alignment, an increased disc height, a higher fusion rate, a lower subsidence rate, and a lower revision rate compared with cage-alone constructs (23). However, new complications associated with anterior cervical plating have arisen, and among those complications, the postoperative dysphagia and adjacent segment degeneration have been the most widely investigated (5–7, 24). Therefore, the primary motivation for developing various “low-profile” or “zero-profile” anchored spacers was to mitigate these plate-related complications while still providing sufficient postoperative stability. It has been widely reported that these devices can reduce the occurrence of postoperative dysphagia by decreasing plate prominence, irritation to the esophagus, and prevertebral soft tissue edema. They also have the potential to reduce adjacent segment degeneration by minimizing stress concentration and damage to adjacent structures (9, 24–26). This widely accepted clinical trade-off provides essential context for our radiological findings and explains why surgeons continue to choose these devices. Consistent with this, our study found that both the Zero-P VA device and the PCC provided significant and comparable improvements in short-term clinical outcomes, as measured by VAS and JOA scores. This aligns with numerous other studies and suggests that the Zero-P VA device can achieve a therapeutic effect comparable to that of the traditional plate-cage construct in single-level ACDF (9, 24, 25).

In this study, we focused primarily on the radiological outcomes, which were reflected by Cobb C, Cobb S, AHS, PHS, rate of segmental kyphosis, and the rate of bone fusion. The cervical alignment plays an essential role in maintaining spinal balance and stability, and reconstruction of cervical physiological lordosis is of critical importance to orthopedists (24). Loss of lordosis or cervical kyphosis is associated with pain, disability, and undesirable shifts in loading of the thoracic and lumbar spine, and may result in adjacent segment degeneration or other issues (27). Rostami et al. examined the changes in segmental and global cervical sagittal parameters after ACDF and found a significant correlation between the increase in Cobb C and Cobb S and the improvement in neck pain and NDI at 1- and 6-month follow-up (28). A prospective, randomized, double-blind clinical study with an average follow-up period of 37.5 months by Villavicencio et al. also indicated that maintaining or increasing Cobb S was associated with greater improvements in clinical outcomes (29). Another study with an average follow-up period of 9.8 years showed that the postoperative kyphotic change in both the cervical spine (represented by Cobb C) and the fused segment (represented by Cobb S) were associated with adjacent segment degeneration after ACDF (30). Moreover, the reduction in Cobb S and Cobb C may lead to compensatory changes in adjacent segments, C1–2 segments, thoracic, and even lumbar regions (28). Therefore, restoration of both Cobb C and Cobb S should be considered in every ACDF as an indicator of improved functional outcome of patients. Our study showed that the Zero-P VA device could achieve postoperative Cobb C correction comparable to that of PCC, which is consistent with previous studies comparing other types of anchored spacers with PCC in single-level ACDF (4, 10, 13, 16, 24, 25). However, regarding Cobb S, our findings differed from the prevailing results of previous studies. Our study demonstrated that Zero-P VA was less effective than the PCC in maintaining surgically corrected Cobb S at 3 and 12 months after surgery, with a statistically significant difference. In addition, the rate of segmental kyphosis in the Zero-P VA group was significantly higher than that in the PCC group (12% vs. 0%). This finding is noteworthy because, although less commonly studied than Cobb C (31) the correction of Cobb S is increasingly associated with better long-term functional outcomes and a lower risk of adjacent segment degeneration (27–30). To understand this discrepancy, it is crucial to examine the literature on its predecessor, the 4-screw Zero-P device, on which most existing research is based. The stability of the original Zero-P has been a topic of discussion. Biomechanical analyses, such as those by Scholz et al. and Reis et al., concluded that the 4-screw Zero-P provided biomechanical stability similar to that of the established PCC, despite slightly more ROM during flexion and extension (6, 32). In clinical practice, this generally translated to comparable radiological outcomes. Most studies suggest that the Zero-P device can maintain Cobb S at a level comparable to that of PCC in single-level ACDF (4, 10, 16, 25). For example, a retrospective study by Noh et al. and another by Zhang et al. both showed no statistically significant difference in postoperative Cobb S between the Zero-P and PCC groups (16, 25). Even studies like Abudouaini et al., which found Zero-P to be less dynamically stable at 3 months, reported no significant difference in the final static Cobb S between the groups (4). Furthermore, a study by Yun et al. indicated that the efficacy of Zero-P and PCC were comparable in maintaining Cobb S, provided the implant was appropriately positioned at the anterior vertebral line (17). Therefore, the prevailing consensus for the 4-screw Zero-P is that, when well positioned, it appears sufficient for the correction and maintenance of Cobb S. Given that the Zero-P VA is a direct evolution of this device, incorporating features like safety stops to aid alignment, one might expect similar or superior performance. However, our study demonstrated a different and inferior outcome in maintaining segmental lordosis. The reasons for this disparity likely lie in the specific design changes of the Zero-P VA and the intraoperative procedures it requires.

We believe that a primary reason for this may be related to the inherent stability of the Zero-P VA device itself. Due to different designs, it is difficult to compare the stability of Zero-P VA and PCC from a design perspective. However, Zero-P VA can be compared with Zero-P, which has a similar design. The main difference between Zero-P VA and Zero-P are that the number of screws has been reduced from four to two, and the type of screws has been changed from fixed angle screws to variable angle screws (33). Panchal et al. reviewed previous biomechanical literature and reported that no-profile devices, despite variations in screw number, typically support ROM reduction comparable to that of a traditional plate and cage construct in ACDF (34). In addition, a biomechanical analysis by Reis et al. showed that the two-screw Zero-P VA demonstrated performance similar to that of Zero-P and PCC within the stiff zone once the hardware was engaged (32). The variable-angle screw design facilitates ease of use and allows postoperative screw toggling within the vertebral bodies, which may potentially prevent or limit load shielding of the bone graft in the event of subsidence (33). However, allowing variable angle screws to toggle postoperatively within vertebral bodies may lead to inferior postoperative stability within the variable angle range of the screws. Reis et al. further demonstrated that the Zero-P VA allowed significantly greater ROM than the PCC during flexion, extension, and axial rotation, and greater ROM than the Zero-P during extension and axial rotation, while no statistically significant differences in ROM were observed among the three groups within the stiff zone (32). A biomechanical analysis of the Zero-P VA and Zero-P under conditions of progressive cervical flexion-distraction injury also showed that the variable angle screw spacer did not sufficiently stabilize flexion–distraction injuries, and suggested that free motion permitted by the variable-angle design may allow the implant to function as a free spacer within this unrestrained range (19). Taken together, existing biomechanical evidence suggests that the stability of the Zero-P VA is inferior to that of both Zero-P and PCC, and that the primary contributing factor appears to be the variable-angle screw design rather than the reduction in screw number. Although the threshold of stability required to maintain satisfactory postoperative Cobb angle remains unclear (35), the stability provided by the Zero-P VA device in our study may fall below this theoretical requirement.

Another important reason for the inferior Cobb S and rate of segmental kyphosis in the Zero-P VA group may be the necessary intraoperative manipulations of the endplates, the anterior osteophytes and the anteroinferior margin of the cranial vertebral body. These intraoperative manipulations may cause some biomechanical analysis studies of zero-profile systems to fail to fully reflect clinical conditions, as the aforementioned biomechanical studies by Scholz et al. and Reis et al. did not incorporate these intraoperative procedures (6, 32). The upper endplates of the cervical vertebral body are dome- shaped, whereas the lower endplates are concave. However, the upper and lower surfaces of Zero-P VA are of non-anatomical design. Before inserting of the Zero-P VA, the endplates are polished to facilitate device placement, which may lead to loss of endplates strength. A biomechanical analysis by Cheng et al. showed that there was a 44% loss of endplate strength even when 1 mm of endplate was polished (36). Because the screws of the Zero-P VA device enter the cancellous bone of the vertebral body through the endplates, a decrease in endplate strength may lead to reduced screw fixation strength and increased cage subsidence. A computed tomography osteoabsorptiometry study by Zavras et al. showed that the bone at the anterior margin of the intervertebral space represented the hardest region contacted by the Zero-P VA device (37). However, anterior osteophytes are removed to ensure that the anterior titanium alloy plate of Zero-P VA can be positioned at the anterior vertebral line, and the anteroinferior margin of the cranial vertebral body is removed when the surgical field of view is obstructed. Both procedures may inevitably reduce the bone strength at the anterior margin of the intervertebral space, particularly the latter, resulting in the exposure of cancellous bone after the cortical bone is removed. The anterior margin of the intervertebral space coincides with the position of the anterior titanium alloy plate, which represents the hardest component of the Zero-P VA. This creates a substantial mismatch in elastic modulus between the anterior titanium alloy plate and the adjacent bone following removal of the anterior osteophytes and the anteroinferior margin of the cranial vertebral body, thereby potentially increasing the likelihood of subsidence at the anterior margin of the intervertebral space. A prospective, randomized, controlled study of a stand-alone anchored cage (PREVAIL) with a 2-year follow-up also emphasized the biomechanical support provided by anterior osteophyte, demonstrating a subsidence rate comparable to that of PCC (38). Another study of Zero-P in 1–3 levels ACDF by Zhang et al. showed that preservation of bone at the anterior margin of the vertebral body can effectively reduce the risk of Zero-P subsidence (39). The study of Zero-P by Deng et al. presented a similar viewpoint, showing that excessive intraoperative manipulation may create a space between the titanium alloy plate of Zero-P and the endplate, which may result in inferior Cobb C, Cobb S, and intervertebral disc height compared with cases without such a space (40). Under axial loading, when the facet joints provide adequate support to the posterior part of the vertebra, it can be inferred that inferior stability of the Zero-P VA during flexion and extension, reduced screw fixation strength, and substantial elastic modulus mismatch at the anterior margin of the intervertebral space may lead to greater loss of anterior height of the surgical segment (AHS), thereby contributing to inferior Cobb S and a higher rate of segmental kyphosis. Our results also showed that AHS at 3 and 12 months after surgery in the Zero-P VA group was lower than that in the PCC group, while the values were comparable immediately after surgery. Our inferior radiological outcomes may be related to these intraoperative manipulations. In contrast, these intraoperative manipulations do not affect the plate and screws of PCC. In patients treated with PCC, physiological mechanical loading counteracts the anterior tension generated by the plate–screw construct, with screws anchored in the anterior cortical bone, leading to an increase in Cobb S during the early postoperative period until the facet joints engage and provide sufficient posterior support. Our results also showed that PHS in the PCC group at 3 months after surgery decreased significantly compared to that immediately after surgery, and Cobb S at 3 months after surgery in the PCC group increased slightly compared to that immediately after surgery, although the difference was not statistically significant (*p* = 0.108). These findings were consistent with the prospective study by Vanek et al. (22) and contributed to the increasing difference in Cobb S between the Zero-P VA group and the PCC group.

Our results showed that Zero-P VA could achieve a satisfactory fusion rate comparable to that of PCC, which was consistent with some previous studies on Zero-P in single-level ACDF (25) and Zero-P VA in three-level ACDF (14). The variable-angle screw design may contribute to the satisfactory fusion rate by potentially preventing or limiting load shielding of the bone graft in the event of subsidence, through a mechanism similar to that of the dynamic plate (41). In addition, the aforementioned study by Deng et al. showed that excessive intraoperative management resulted in a quicker fusion process due to more contact and compression between the bone graft in the posterior PEEK cage and endplates, although those managements led to inferior stability (40). Micro-motion may stimulate bone formation within the ACDF segment, while also contributing to implant subsidence before fusion (42).

Our study has several limitations. First, its retrospective design prevented randomization and is subject to selection bias. Second, as a single-center study, its results may not be generalizable to other institutions with different surgical practices. Third, the follow-up duration of 12 months is relatively short. While sufficient to observe early radiological changes, it is not long enough to determine if these differences in segmental alignment may translate into differences in long-term clinical outcomes, such as chronic neck pain or the incidence of adjacent segment disease. Thus, further multi-center, prospective, randomized controlled studies with long-term follow-up are needed to confirm our findings and evaluate their long-term clinical significance.

## 5 Conclusion

Compared with the PCC, the Zero-P VA device provided comparable clinical outcomes in single-level ACDF at the 12-month follow-up. However, regarding radiological outcomes, the Zero-P VA device resulted in an inferior segmental angle, anterior height of the surgical segment, and a higher rate of segmental kyphosis. These findings may be associated with the inherent biomechanical properties of the two-screw, variable-angle device and the necessary intraoperative preparation of the endplate, as well as removal of the anterior osteophytes and the anteroinferior margin of the cranial vertebral body. While short-term clinical results were not affected, the long-term consequences of this loss of segmental lordosis remain unclear. Therefore, the choice between these two implants represents a clinical trade-off: surgeons must weigh the established benefits of a zero-profile device, against the superior ability of the PCC to restore and maintain segmental lordosis.

## Data Availability

The original contributions presented in the study are included in the article/Supplementary Material, further inquiries can be directed to the corresponding author.
